# Hospital nurses and physicians’ experiences practicing patient safety work to recognize deteriorating patients: a qualitative study

**DOI:** 10.1186/s12913-024-11908-x

**Published:** 2024-11-18

**Authors:** Astrid Marie Nysted Berg, Anne Werner, Ingrid Ruud Knutsen, Anne-Kari Johannessen

**Affiliations:** 1 Department of Nursing and Health Promotion, Faculty of Health Sciences, OsloMet – Oslo Metropolitan University, P.O. Box 4, St. Olavs Plass, Kunnskapsveien 55, 2006 Kjeller, Oslo, Norway; 2 Anesthesia Department, Akershus University Hospital (Ahus), P.O. Box 1000, Lørenskog, N-1478 Norway; 3 HØKH – Health Services Research Unit, Akershus University Hospital (Ahus), P.O. Box 1000, Lørenskog, N-1478 Norway

**Keywords:** Patient safety, Deteriorating patients, Rapid Response Teams, Health professionals, Collaboration, Competence, Competencies, Empowerment, Qualitative study

## Abstract

**Background:**

Measures to increase patient safety work aim to prevent patient harm and injuries in hospitals and are a priority worldwide. Several hospitals have implemented various rapid response systems to strengthen patient safety. Knowledge is limited concerning how health professionals interact and how they experience competence development and master emergency situations. The aim of the study was to explore and describe hospital nurses’ and physicians’ experiences with patient safety work, and the implications of this work for collaboration and competence in a hospital.

**Methods:**

We used a qualitative descriptive design and interviewed 21 nurses and physicians from a hospital in Norway. Data were analysed with systematic text condensation, a four-step thematic cross-case analysis. The study adheres to the COREQ guidelines for conducting qualitative research.

**Results:**

Through analysis, three categories were identified: strengthening a common clinical language for patient deterioration; sharing and transferring competencies across hospital wards and amongst professionals; and balancing time pressure and limited resources. The results showed that the patient safety work contributed to building bridges between health professionals and across hospital levels regarding deteriorating patients. It also provided structure and systematization to the hospital professionals’ work with deteriorating patients. However, the results also demonstrated the complexity of maintaining patient safety, pointing to the need to balance limited time and resources in hospital.

**Conclusions:**

The patient safety work presumably provides better emergency care, which may enhance patient safety in hospital. The complexity of hospital work, however, can influence the patient safety performance negatively, requiring health professionals to adopt robust, up-to-date skills and competencies in observing and assessing patient deterioration. In a busy hospital setting, the ability of health professionals to collaborate and communicate is important as they are faced with the challenges of balancing time-pressure and limited resources, which can potentially compromise patient safety work.

**Supplementary Information:**

The online version contains supplementary material available at 10.1186/s12913-024-11908-x.

## Background

Over the past century, the demography in Northern Europe has changed considerably, thus increasing the number of emergency admissions and admissions of critically ill patients in hospitals [2, 3]. Particularly the number of older and more fragile patients is expected to increase in the years to come [4]. More inpatients with complex disorders and comorbidities place greater demands on health professionals’ competence and performance in healthcare services [3, 5, 6]. Over the past 15 years, studies that map undesirable incidents and patient injuries in hospitals in high-income countries have shown a disturbingly high incidence of around 10–12% [4]. Typical patient injuries include hospital infections, unsafe care, technical complications from surgery, drug-related injuries and blood clots that increase morbidity and mortality [2, 4, 6, 7]. Patients exposed to harm in medical care are a public health concern which prolongs the average length of hospital stay, permanent disability or even death in up to 10% of inpatient cases [6, 8].

A report from the Organization for Economic Co-operation and Development (OECD) suggests that investments in limiting patient harm can reduce the burden of injury up to 15% and improve patient outcomes, as well as reducing costs in health care systems [9]. A policy goal in Norway and worldwide is to reduce or prevent patient harm and injuries by focusing on measures to increase patient safety work in healthcare [3, 10]. The World Health Organization (WHO) defines patient safety as:

a framework of organized activities that creates cultures, processes, procedures, behaviors, technologies and environments in health care that consistently and sustainably lower risks, reduce the occurrence of avoidable harm, make errors less likely and reduce the impact of harm when it does occur. [11]

Challenges related to patient safety in hospitals are a complex matter and have led to a need for increased competence [6, 12]. Transitions within and between health service levels, relationships between specialities, shifting tasks and changed patient flow together with shorter length of hospital stay are associated with risk and a challenge to patient safety [5, 13]. Decades of research have established that early identification of patient`s deterioration together with rapid and effective response to a condition can mitigate adverse events, health disease progression, and reduce the incidence of cardiac arrest [6, 14].

### Rapid Response Systems— a patient safety package

Patient safety work aims to prevent and reduce risks, errors and harm through organized activities to reduce the severity of unsafe care, such as strengthening cultures of safety procedures, behaviours and various medical or monitoring technologies [10, 15]. The work includes learning and understanding the use of standardised tools to prevent and treat life-threatening conditions. Rapid Response Systems (RRSs) are an organization’s “safety net” for protecting deteriorating inpatients and involves advice to health professionals to help them identify and improve recognition of and response to inpatients outside of the intensive care unit [15]. Over the past 30 years, hospitals worldwide, including in Norway have adopted RRSs in clinical practice, implementing them in a variety of ways, to suit the hospital`s specific needs and contexts.

The Norwegian Directorate of Health recommends implementing an extended “patient safety package” within a hospital setting [16, 17]. The extended patient safety package includes an educational programme, training health professionals in a proactive course (proACT), which includes the risk assessment tool of the National Early Warning Score (NEWS) [18], and systematic measures of vital parameters namely: Airway, Breathing, Circulation, Disability, Exposure and Further care (ABCDEF) [19]. The NEWS tool is intended to complement clinical judgement when assessing a patient`s condition by checking six vital physiological parameters: respiratory rate, oxygen saturation, temperature, systolic blood pressure, pulse rate and level of consciousness [8, 20]. The patient safety package also includes the communication tools of Identification, Situation, Background, Assessment and Recommendation (ISBAR) [21], representing a common communication code standing for essential elements in the patient safety work and the assistance from the Rapid Response Team (RRT) [3, 4, 21, 22]. Both ABCDEF and NEWS are priority-based approaches designed for recognition, assessment and triggering of responses, and treatment of deteriorating patients referred to as afferent limb activities [19, 20, 22, 23]. In this patient safety package, the efferent limb activities refer to the assistance from the RRT [3, 4, 24]. The terms RRS and the outreach teams, such as the RRT and medical emergency teams (MET), are often used interchangeably [15]. In this study, we refer to the RRT as an outreach team, including both an intensive care nurse and a physician, on call within the hospital setting. Furthermore, the terms “patient safety package” or “safety package” refer to the specific RRS – including RRT and a proACT course.

### Collaboration and competency transfer between hospital nurses and physicians to strengthen patient safety

According to WHO [4], the best effect of RRSs is when education and training ensure the right competence of health professionals together with monitoring patients’ vital functions (NEWS), recognition of worsening condition, and an adequate response (RRT) that ensures quick help when a patient’s condition is deteriorating. Despite positive experiences with RRT, studies point out that the efficacy of an RRT varies in improving a patient’s outcome [14, 15, 25]. Nevertheless, studies also show that RRTs, which to a great extent are nurse-led services, imply more effective patient care and improved patient outcomes when involving physicians by systematizing their clinical work [2, 26, 27]. Furthermore, a scoping review by Wood, Chaboyer and Carr [28] identifies obstacles to the use of NEWS for health professionals in hospitals, following a lack of trust across hospital wards and professionals. Alam et al. [29] conclude in their review that the NEWS measurements are easy to apply bedside coupled with a hospital outreach team, such as an RRT. Still, many nurses hesitate to call the RRT due to fear of being criticized by the specialized response team [28, 30].

Ward nurses have a substantial role in the improvement of quality patient care and patient outcomes [31]. A study by Dougherty and Larson [32] refers to collaboration between nurses and physicians, and points out that communication and shared decision-making among nurses and physicians contribute to a decrease in errors and morbidity for patients. Studies have reported interprofessional tension and uncertainty regarding individual responsibilities with a lack of role clarity, blurred professional boundaries, increased burden on hospital RRT members and tension between the nurses and the physician in the teams [24, 31, 33]. Patient safety is highlighted as a priority area in constant need of collaborative practice and training for further improvement [10, 34].

In a review of how hospital health professionals recognize and respond to patient deterioration, Allen, Elliott and Jackson [24] argue that it is necessary to develop a better understanding of interprofessional collaboration and how professionals can best work together to quickly identify and respond to acute worsening in the condition of patients. The research literature has also pointed out the need to look more closely at what happens in social learning processes in health services to gain an understanding of how health professionals engage in this work [35]. Drawing from Kanter’s theory of structural empowerment [36], the quality of patient care within a teamwork setting can be understood to depend on empowered health professionals performing new skills, particularly amongst newly educated health professionals. Her theory within organizations is associated with employees’ access to information, resources, support, and problem-solving capabilities within a supportive interdisciplinary team [36]. Researchers have expanded upon Kanter's theory of structural and individual empowerment to encompass the role of the employer and the opportunity for growth, learning, and mastery in patient safety learning environments [37, 38]. The perspective of Kanter on empowerment may offer a useful approach to understanding the experiences of hospital professionals in relation to the applicability of the patient safety package. To our knowledge health professionals’ experiences of applying the extended patient safety package has to a limited degree been explored in a Norwegian hospital context.

## Methods

### Aim

The aim of the study was to explore and describe hospital nurses’ and physicians’ experiences with patient safety work. We focused on implications of this work of using an extended patient safety package for collaboration and competence in a hospital.

### Design

We conducted a qualitative interview study with a descriptive design [39], aiming to explore experiences as expressed by the health professionals themselves. We applied the checklist guidelines for the Consolidated Criteria for Reporting Qualitative Research (COREQ) in preparing the manuscript [40].

### The study setting and the educational programme

The study was conducted at a university hospital in Eastern Norway, one of the few Norwegian hospitals that has implemented an extended patient safety package, including RRT and a proACT course. All health professionals working with patient care must complete an educational programme within their first year as a hospital employee. The programme includes a proACT-course consisting of theory and simulation training on the clinical use of the safety tools of NEWS, ABCDEF, and ISBAR. This educational programme is implemented in all general medical and surgical wards in the hospital. All adult inpatients are routinely measured during their hospitalisation according to the NEWS score from low to high. A higher score indicates an increased risk of clinical deterioration.

The health professionals use the NEWS measures together with their concern about the patient’s clinical condition, to consider when it is necessary to escalate to further care and contact ward physicians or the RRT. The RRT was established at the hospital in 2016, as an outreach team for all the general medical and surgical wards except neonatal intensive care. It consists of an intensive care nurse and an anaesthetist or medical physician on call from the intensive care unit to assist health professionals in the wards every day around the clock. The purpose of the RRT is based on the prospective identification of high-risk patients by health professionals in the hospital wards giving a notification at an early point to the response team. In addition, RRT professionals are trained to carry out an intervention within 20 min to acutely ill ward patients.

### Recruitment and data collection

A user involvement process including a user panel with participants representing patients, hospital leaders, nurses and physicians informed the aim of the study. Information about the study was distributed to all general medical and surgical wards and RRT members in the hospital. A purposive sampling strategy was used, covering nurses and physicians, representing different ages, genders, medical specialties, and lengths of experience in hospital work using the RRT or being part of the RRT [39]. Interested physicians and nurses reported to their leaders who then forwarded their contact information to the first author. In the inclusion process many showed interest in participating.

We carried out in-depth interviews with 21 nurses and physicians who had experience with the patient safety package. Recruitment was concluded once we had assembled a diverse group of participants with a wide range of experiences. The first author conducted the interviews between January and October 2021 in the hospitals’ facilities. Lasting between 30 and 60 min, each interview was digitally recorded and transcribed verbatim by the first author. All interviews were open-ended and based on a semi-structured interview guide which had been developed in advance [41]. The guide was, based om the aim of the study, focusing on collaboration and competence surrounding the participants’ experiences of the patient safety package. The research team developed the questions and tested them with a pilot interview. The participants were asked different questions based on whether they were members of the RRT or ward professionals, about their experiences with training and usage of the safety package. Furthermore, the ward professionals were asked about their experiences with calling RRT, including learning from and collaboration with the response team across health professionals and wards. Similarly, the participants from the response team were asked about their experiences of collaboration with professionals in the wards when being called on by nurses or physicians due to deterioration in a patient’s condition.

### Data analysis

Transcripts of the audiotaped interviews constituted the data for this study. The data material was analysed using systematic text condensation (STC), a cross-case method for thematic analysis developed by Malterud [39]. During the coding process, all authors routinely met every other week for about a year and negotiated the four steps of analysis. The first step included reading all the data material to identify preliminary themes and obtain an overall impression of the participants’ experiences from applying the safety package. In the second step, meaning units were coded – i.e. identified, classified, and sorted – representing various aspects of the participants’ experiences of and challenges in relation to the safety package. Throughout the data collection and the analysis, it became obvious that the hospital health professionals were concerned about patient safety. They also revealed various experiences and different dilemmas regarding the safety package and RRT. During the coding process, our focus was on health professionals’ collaborative response to deteriorating patients’ conditions and the significance of the safety package in clinical work for collaboration and competence transference. In the third step, condensation from code to meaning with subgroups was established. Subgroups represented essential aspects of each code group across the participants, condensing the meaning content and selecting quotations that in a suitable way illustrated the essence of the participants’ descriptions. Finally, the condensates were synthesized to form a generalized description that reflected the main results of hospital professionals’ experiences of the implications of the patient safety package.

### Reflexivity

The research group included a medical sociologist and three nurses, amongst whom was an intensive care nurse and a nurse anesthetist. Three of the authors were experienced in qualitative methods and were currently working as academics and researchers. The first and the last author, both nurses, have many years of experience in clinical work and patient safety work within hospital settings. Additionally, the first author is a member of the hospital’s RRT. Proximity to the research field may be a challenge. The first author’s preconceptions were brought into the study of experiences, hypotheses, and professional and theoretical perspectives, which included an understanding that the RRT is an important team in the hospital, contributing to collaboration and competence around deteriorating patients and aiding wards in saving lives. The challenges of proximity provided critical reflection and awareness from all authors. Reflexivity is an important part of qualitative analysis [39]. During the data collection, the research group discussed critically the data collected and the interviews, challenging potential preconceptions. In accordance with the analytic procedure of Malterud [39], stepwise analysis during data collection enhanced the possibility of sharpening focus and aims and also of enabling a process of intersubjectivity and reflexivity, while maintaining a responsible level of methodological rigour. The variety in the research group’s backgrounds might have made up for the first author’s proximity to the research field.

## Results

The sample included four physicians, and seventeen nurses aged between 21 and 69 years old. Their hospital work experience ranged from 6 months to 20 years. Twelve of the participants – two physicians and ten nurses – were hospital ward professionals, while nine of the participants, i.e., two physicians and seven nurses, were part of the RRT (see Table 1).

**Table 1:** Descriptives of the study participants (*N* = 21)

**Age**	***n =***
20–29	4
30–39	7
40–49	5
50 +	5
**Gender**	***n* = **
Men / Women	4/17
**Speciality**	***n* = **
Physician, RRT	2
Nurse, RRT	7
Nurse, surgical ward	4
Nurse, medical ward	4
Nurse, women`s clinic	1
Nurse, orthopaedic ward	1
Physician, surgical ward	1
Physician, medical ward	1
**Length of experience (years) in hospital**	***n* = **
0–5	7
6–10	5
11–20	3
21–30 +	6
**proACT course**	***n* = **
Participated	18
Did not participate	3

Through analysis, the research group identified three main categories in the interviews: (1) strengthening a common clinical language for patient deterioration; (2) sharing and transferring competencies across hospital wards and amongst health professionals; and (3) balancing time pressures and limited resources (see Table 2).

**Table 2:** Examples of the analysis process

Meaning units—preliminary themes	Code groups	Subgroups	Categories
*Using the NEWS terminology is a natural part of the daily vocabulary and a starting point for dialogue between colleagues about a deteriorating patient. (Ward physician, 18)* *We come out to the ward and see that they are too concerned with measuring the patient’s blood pressure or something else. The nurses do not see that the patient doesn’t have free airways and therefore has a snoring respiration, and I think that is very scary. Then our team, accordingly, arrives in the wards and takes over due to a lack of competence. (RRT nurse, 2) *	The strengthening of a common structured language terminology including NEWS, ISBAR and ABCDEF Practical patient safety tools to assess the patient’s condition It is a risk to rely too much on NEWS scores at the expense of the clinical gaze	Strengthening and learning a common structured clinical language for patient deterioration Strengthening and sharing competence on an individual and on a system level	Strengthening a common clinical language for patient deterioration
It’s good that we from RRT come, because we can assist each other and help to move the deteriorated patient to a higher treatment level. (RRT nurse 2) One time when the ward physician did not answer me, and I was alone with a severely ill patient, I called the RRT and told them that I needed to get medical supervision. The physicians had not answered, and I needed help with a deteriorating patient. In that situation, I remember, I received the honour of having the RRT physician say that I had done exactly right, and that this was a patient who earlier should have been transported to an intensive care unit. Afterwards, I was satisfied with having called RRT. (Ward nurse 12) The ward nurses have more than enough responsibility with far too many ill patients and often the absence of the physicians on duty. The ward physicians are often in the operating room and give advice over the phone, which can be to contact RRT. Then the nurses call us, and we come to the ward. Then, we claim that the ward physicians should attend to their deteriorating patient. (RRT nurse, 1)	Learning interdisciplinary competency and transference You must be decisive to manage the complexity of acute life-threatening episodes in hospitalized patients Interdisciplinary collaboration and sharing competencies as a learning arena around the patient’s bed Severely sick patients imply substantial support from RRT	The significance of interdisciplinary collaboration, sharing and transferring competencies across wards and between health professionals RRT has a mentoring and educational role RRT acknowledge they have considerable responsibility for supporting the ward professionals	Sharing and transferring competencies across hospital wards and between health professionals
*Although it is much more structured to work after NEWS, it is also challenging. If the patient scores a low NEWS, then you must follow the guidelines and score the patient after a few hours again. It is hectic to prioritize several NEWS scores on a patient whose oxygen level is stable at 95%, when the ward is busy* *and there are many other fragile patients. (Ward nurse 8)* *It is a tough ward to work in, with many critically ill patients we are responsible for, probably several critically ill patients at the same time. The amount of experience we have varies. (Ward nurse 12)*	There is time pressure, and it is time-consuming to measure a NEWS score Stressful situations for ward nurses standing bedside alone with severely ill patients	NEWS is a time-consuming procedure Heavy workload and responsibility with high staff turnover and many newly graduated nurses The danger of RRT becoming a replacement team substituting the absence of physicians	Balancing time pressures and limited resources

### Strengthening a common clinical language for patient deterioration

The results showed that both nurses and physicians underlined how the patient safety package contributed to a unifying clinical language of communication across hospital wards and the RRT and between health professionals as well. The participants experienced the idea that ISBAR and NEWS helped them to learn and structure the communication so that the message of a patient’s deterioration could more quickly be conveyed between the health professionals. When professionals in the wards knew and managed this communication terminology, it became easier to achieve a mutual review and understanding in each patient’s case. As one ward physician said:

Using the NEWS terminology is a natural part of the daily vocabulary and a starting point for dialogue between colleagues about a deteriorating patient. (Ward physician, 18)

Many ward nurses and physicians emphasised the significance of learning to structure the work by using NEWS, and ABCDEF expressed more precisely their concern about a patient when calling for help. As one of the ward nurses shared:

I feel that the tools are a supplement, because as a new graduate nurse you are a bit on your own. Last time I checked on a sick patient I was thinking, what can I do now? Then I thought I can measure a NEWS and if it is high, then I can call for further help. (Ward nurse, 14)

The ward nurses said that mastering and using both NEWS measures and ISBAR had strengthened their own competence and confidence in how and what to communicate to the ward physician and when to call the RRT. One of the ward nurses said:

Earlier, I could call the physician and say that I have a deteriorated patient with fever and not much more. Now I report on the NEWS score and with slightly more information of vital functions if the patient has a very high NEWS score. When I called before, the physician wouldn’t come right away, but now I think they follow up patients with a high NEWS score more closely. (Ward nurse, 12)

The ward nurses highlighted how much easier it was to get the physician’s attention when referring to NEWS measures than trying to describe “a negative gut feeling” regarding the patient’s condition. Now they had the vital measures to add to the conversation. However, despite all the positive experiences with applying the common clinical language of patient safety, the RRT professionals mentioned the danger of becoming overly preoccupied with conveying and paying attention to NEWS values. Both the RRT nurses and physicians emphasised the risks that the ward nurses would rely too much on the NEWS measures alone at the expense of “one’s own clinical gaze.” One RRT nurse said:

We come out to the ward and see that they are too concerned with measuring the patient’s blood pressure or something else. The nurses do not see that the patient doesn’t have free airways and therefore has a snoring respiration, and I think that is very scary. Then our team, accordingly, arrives in the wards and takes over due to a lack of competence. (RRT nurse, 2)

### Sharing and transferring competencies across hospital wards and between health professionals

Both ward professionals and RRT members reported that the competence within the teams and the collaboration across hospital wards were strengthened due to applying the tools for observation and risk assessment of patient deterioration. The ward nurses also described how the RRT professionals made them feel calmer and safer when providing help, especially when the ward physicians were unavailable because they, for instance, were performing surgery on patients. The answers of the participants being interviewed indicated that the safety package created a learning arena around the patient’s bed where ward professionals having varied experience as health professionals together with the RRT could safely acquire practical skills to master to assist deteriorating patients. One of the ward nurses talked about the positive experience of calling the RRT after an agreement with the ward physician about a patient who had a high NEWS level:

I remember an RRT nurse who came and helped me hang up the fluid. We started with oxygen and initiated measures. She called me up and came over during the night without me asking – just to follow up measures we had started on. I felt that I got so much help – that I was not alone in the situation. (Ward nurse, 17)

The ward nurses and physicians also told us how applying NEWS as a daily routine felt like helping and mastering their clinical judgement, making it easier for them to prioritize amongst patients who they assessed needed support from the RRT. On the other hand, the RRT participants pointed to how they had exerted themselves to share clinical expertise when they were called on by the ward’s professionals. Both ward and RRT professionals called attention to how interdisciplinary collaboration was decisive in managing the complexity of acute life-threatening episodes in the lives of hospitalized patients. One of the RRT nurses thought the team represented important support and supervision and said:

It’s good that we from RRT come, because we can assist each other and help to move the deteriorated patient to a higher treatment level. (RRT nurse 2)

Many of the RRT professionals emphasised the mentoring and educational role of their work in relation to colleagues in the wards, enabling them to master emergency situations through the safety package. The ward physicians and nurses described the educational aspect of RRT as they provided reassuring medical assessments and confirmation of the situation. All the participants identified assistance with decision-making support and rapid clarification on further treatment or treatment level as important bedside learning exercises for the ward professionals.

The ward professionals described how they used the RRT’s expertise to assess whether a worsened patient needed to be transferred to a higher treatment level. All the ward nurses emphasised that they often were alone with responsibility for severely sick patients. The ward nurses underscored that they experienced the ward physicians as being difficult to reach. That was an important part of why they approached the RRT to get assistance regarding deteriorating patients. RRT nurses and physicians reported that many of the hospitalised patients were severely ill, and they realized how important it was to rapidly assist the wards when they called the team. A ward nurse talked about her experiences:

One time when the ward physician did not answer me, and I was alone with a severely ill patient, I called the RRT and told them that I had tried to get medical supervision. The physicians had not answered, and I needed help with a deteriorating patient. In that situation, I remember, I received the honour of having the RRT physician say that I had done exactly right, and that this was a patient who earlier should have been transported to an intensive care unit. Afterwards, I was satisfied with having called RRT. (Ward nurse 12)

Some of the RRT professionals noted that they sometimes had to “claim” that not only the ward nurses but also the ward physicians should participate when the RRT supported the wards. One of the RRT nurses reflected on having the responsibility for supporting the ward professionals:

The ward nurses have more than enough responsibility with far too many ill patients and often the absence of the physicians on duty. The ward physicians are often in the operating room and give advice over the phone, which can be to contact RRT. Then the nurses call us, and we come to the ward. Then, we claim that the ward physician should attend to their deteriorated patient. (RRT nurse, 1)

### Balancing time pressure and limited resources

Despite all the positive aspects emphasised by the professionals in relation to patient safety work, the ward nurses also described how time-consuming it was to measure NEWS several times a day for all inpatients. This represented yet another chore in a ward that was already under time pressures. They referred to concurrent time conflicts with follow-up talks with caregivers and supervision of students and new colleagues. One participant said:

Although it is much more structured to work after NEWS, it is also challenging. If the patient scores a low NEWS, then you must follow the guidelines and score the patient after a few hours again. It is hectic to prioritize several NEWS scores on a patient whose oxygen level is stable at 95%, when the ward is busy and there are many other fragile patients. (Ward nurse, 8)

Ward professionals described how it felt having to bear a heavy responsibility for fragile patients alongside other workload tasks in the wards. They explained the situation, referring to an understaffed workforce due to high turnover, prioritizing between demanding tasks, having limited resources, and frequently facing overcrowded wards. Both ward nurses and RRT professionals referred to episodes in which the ward nurses had called the RRT as a sort of relief for easing their workload and almost begged them to take the patient with them. As one ward nurse said:

There were only three of us at work, so you are very alone with severely sick patients. Incidents happen with the patients, and you reflect about whether you could have done something differently or would have benefited from talking with experienced professionals and then learn from it in case a similar situation arises another time. (Ward nurse, 17)

Most of the ward nurses emphasised that high turnover entailed having to frequently train newly employed nurses. This also implied that the newly hired nurses often were trained by inexperienced nurses. One of the ward nurses stated:

It is a tough ward to work in, with many critically ill patients we are responsible for and probably several critically ill patients at the same time. The amount of experience we have varies. (Ward nurse, 12)

Several of the ward nurses expressed how exhausting it was with several ill patients to care for, and particularly when ward physicians were busy elsewhere and lacked the time to respond to the ward nurses’ request for assistance. They thought it was stressful to stand alone at the bedside waiting for the ward physician when they needed help. In addition, they experienced insecurity not having physicians to discuss medical solutions with and instead had to wait a long time for a ward physician to authorize transfer of a deteriorated patient to a higher treatment level. The RRT participants sometimes felt that the reason for calling the RRT was the indirect need from the wards to transfer the patient to a higher treatment level:

This is often why the nurses call – because they have tried to get hold of their ward physician – for example, the surgeon – but the surgeon is operating and cannot come –or because it is a little more difficult to get help from the ward physician to get the approval to transfer the patient. (RRT nurse, 6)

The RRT physicians and nurses experienced their role in the response team as an overwhelming task, implying responsibility for rapid decision-making and coming on top of their clinical work in the intensive care and anaesthetic units. The RRT nurses and physicians reported that many of the hospitalised patients were severely ill, and they realized how important it was to rapidly assist the wards when they called the team. Both ward physicians and nurses called the RRT, knowing the assignment often was time-consuming for everyone in the extended team. An RRT physician emphasized that they worked in a persistently challenging work situation, with multiple calls and work demands requiring constant attention:

Sometimes I know that I’m in too much of a hurry and I can’t respond to the RRT call in a reasonable amount of time even if the RRT function is my responsibility. Then I consult with another anesthetist or enquire whether the intensive care nurse can go alone to the RRT assignment. (RRT physician, 21)

Several of the RRT participants described the hazards of their RRT function when they had to act as a substitute for an absent physician. The RRT participants implied that when a patient was deteriorating, their function was to act as a “replacement team” for the ward physicians and nurses. The RRT expressed their reliance on ward physicians and nurses for subsequent treatment decisions, due to their limited patient interactions. The RRT physicians, feared that the support of the response team could lead to a kind of complacency within the wards, thereby impeding their improvement for the wards. They described particularly the absence of the ward physicians, leaving all the responsibility for severely ill patients to the RRT.

However, despite the time-consuming procedures that working with NEWS scores and calling for the RRT entails, ward professionals emphasised that the safety package was an important support and help in their clinical work to detect early deterioration of inpatients. Nevertheless, many of the participants pointed out the need for re-certification of the proACT course and more frequent simulation training in their wards together with the physicians and the RRT, even though this implied yet more work tasks and increased use of their resources.

## Discussion

The first presentation of the results from the present study illustrates the complexity of the patient safety work from the perspective of health professionals in a hospital setting. The health professionals described the importance of adding tools to structure clinical work and strengthen their individual competences in recognizing, responding to, and re-evaluating patients at risk of deterioration. A unifying clinical language contributed to structuring communication in the patient safety work. The results indicated that the sharing and transfer of competencies between health professionals fostered a learning environment surrounding the care of deteriorating patients. However, the results also demonstrated the complexity of balancing time pressures and limited resources in a hospital setting, which represented a challenge to the patient safety work.

### Strengthening individual and structural competence through collaboration in patient safety work in the hospital

We are not the first to report that health professionals have experienced how patient safety work in various ways can contribute to increased competence and collaboration among health professionals [26, 30, 38]. The safety package enables health professionals to structure and systematize their work around a deteriorating patient by using NEWS, ABCDEF and ISBAR. Use of these tools require learning a common language and a structured terminology, which implies a mutual understanding across hospital wards and higher treatment levels. Our results illuminate the importance of a proACT course to gather and train health professionals across specialties to optimize the use of these tools.

Findings from previous studies emphasised that nurses and physicians need to be more aware of the importance of vital signs deviating from several hours ahead of clinical adverse events so they can identify symptoms of deterioration at an early stage [2, 24]. Several studies refer to the importance of outreach teams like an RRT that can provide help in mastering demanding work tasks with deteriorating patients [6, 26, 30]. Earlier research has shown that collaboration barriers such as hospital cultures, workloads, inexperienced staff and weak implementation processes of patient safety tools inhibit effective recognition and response to clinical deterioration [24, 28]. In addition, local needs, various education programmes and resources are found to dominate learning processes of collaboration and competence [13, 28]. Nevertheless, our study points out factors such as balancing time pressure, high turn-over and limited resources that inhibit full utilization of the safety package.

The results of our study show how interdisciplinary collaboration between the RRT, and the ward professionals can enable management of complex and acute life-threatening episodes in hospitalized patients by learning to use an extended patient safety package. Our results add to the existing research emphasizing the importance of focusing on both individual and organizational levels of empowerment in collaboration and competence-building in a hospital setting. We believe that the perspectives of Kanter [36] on empowerment are useful for understanding how learning, collaboration, sharing and transference of competencies between health professionals on wards and across treatment levels happens. Below, we discuss this further and the strengths and limitations of the study.

### The transfer of competencies across hospital levels and between health professionals facilitates empowering processes

Our results illustrate how the RRT can perform as a role model for the ward professionals by assisting them in employing the safety package to communicate and care for critically ill patients. This could potentially foster a sense of empowerment among health professionals. Calling on the RRT for assistance implied offloading and release of ward resources. Other studies refer to positive results of RRTs such as increased knowledge, confidence and clinical performance, real-time redistribution of workload and immediate access to expert help to strengthen health professionals in their teamwork [42, 43].

According to the results of our study, the safety package helped the hospital professionals improve the process of prioritization, which may well increase patient safety work. The collaboration between ward professionals and the RRT bridged a competence gap between the individual health professionals and across hospital wards. This might be understood in line with the concept of organizational empowerment outlined by Kanter [36]. According to Kanter [36] organizational empowerment implies both structural and personal empowerment. This concept underlines the importance of creating a meaningful work environment involving empowerment of nurses and physicians at the workplace level, which is important in supporting patient safety. Kanter [36] refers to structural empowerment as interactional justice, respect and trust that empowers employee job satisfaction and organizational commitment. One study refers to structural empowerment through safety packages as an important component for retaining nurses, preventing burnouts, giving better ability to fulfil job demands in an effective manner and improving job satisfaction and commitment to the organization [37]. Richardson and Storr [38] underscore how nurturing nursing empowerment, leadership and collaboration within the hospital organization can strengthen and support the nurses’ role in patient safety work. Their research highlights that individuals who are sufficiently empowered within the hospital culture can enhance collaboration, owing to their ability to mobilize resources and knowledge that influence the behaviour of their colleagues.

In our study, we found that the RRT supported the ward professional’s bedside by transferring acutely ill patients from the wards to higher treatment levels. The ward professionals also emphasized being empowered by having someone listen to their concerns and trusting their decision about wanting the patient to be transferred. The RRT members collaborated with the ward professionals by providing, e.g., guidance, prioritizing tasks, follow-up patient care, clinical judgement, and decision-making. Other studies have shown that engagement from the RRT is crucial for patient rescue, rapid assessment, basic stabilization, revaluating the situation, providing critical care and making arrangements to move the patient to a higher treatment level [14, 31].

According to Kuşcu Karatepe and Türkmen [12] health professionals who feel empowered and competent within adequate work settings display a positively higher performance of competencies and teamwork than less empowered nurses. The quality of the collaboration may impact the ability to learn and achieve deeper insights and knowledge. When health professionals become more competent, it can encourage and improve further collaboration in the hospital. If the ward professionals experience security and support, even if the situation in the ward is hectic, one might assume that this strengthens the professional’s clinical judgement around the patient’s bed and additionally makes learning easier. Studies show that positive experiences of strengthening patient safety work involve the importance of having a fully functional RRT that can assist, support and guide the ward professionals with early interventions, identification and measures of vital signs and adequate treatment level to improve patient outcomes [6, 26].

### The complexity of balancing patient safety work

Despite the many benefits of the safety package, the results of our study also suggested that there were many contemporary conflicts or various clinically demanding tasks that challenged health professionals in dealing with and recognizing an adverse event in hospitalised patients. A previous study has described hospital physicians’ experiences of being squeezed between different tasks having limited capacity, while attempting to balance patient prioritization and maintaining an efficient patient flow [44]. Due to several hospitalised patients suffering from serious and complex diseases, it is important that health professionals have solid competence. In our study, balancing time pressure and limited ward resources, along with demanding tasks, low ward staffing, high turnover, and few experienced nurses on duty at the same time challenged patient safety. This indicates the importance of investing resources in further developing and implementing the extended patient safety package in the hospital.

Amongst these tools, our study shows that the NEWS measures were specifically helpful to newly graduated ward nurses and physicians when they experienced standing alone with difficult assessments. NEWS became an important tool for further dialogue with RRT and a way to catch the physician’s attention early. However, the ward nurses experienced the use of NEWS as time-consuming. In addition, RRT nurses were concerned that a unilateral use of NEWS would compromise the ward nurses’ clinical judgement. Our study implies that health professionals’ clinical competence is not maintained or prioritized because there is limited time to attend the proACT course, stay updated or find time to train together. The proACT course, engaging staff from inexperienced to experienced, indicates the need for re-certification. The course can foster structural empowerment, leading to improved skills and a positive approach towards collaboration. This structural empowerment aids in transferring competencies and promoting knowledge-sharing within the organization, thereby enhancing the quality of patient safety work in the hospital.

Our findings highlighted the importance of planning for further care to better prioritize hospital resources and showed that the RRTs assisted ward professionals in decisions about which patients would benefit from intensive care and when more conservative care was appropriate. The findings also revealed that RRT physicians were particularly burdened with several overlapping and time-consuming tasks and therefore did not prioritize a proACT course. This challenges the transfer of competencies across the wards and may hamper the effectiveness of patient safety work in the hospital. Other studies have uncovered similar barriers to transference of competencies in hospital settings [14, 31]. According to some studies, patient safety work implies a considerable burden being placed on RRT members and increased tension between nurses and physicians [15, 24, 25, 28].

### Strengths and limitations

This study explores and describes hospital professionals’ experiences of applying a certain extended patient safety package. Our aim was neither to evaluate the patient safety work in the hospital nor to judge the extended safety package according to whether it was better or worse than other rapid response systems. Here, we took a closer look at the implications of this initiative for health professionals’ experiences of collaboration and competence-building in a hospital. To strengthen the relevance of the study a user panel representing patients and hospital health professionals participated in developing the research questions explored in this study. Our preconception from the beginning was that the experience of hospital professionals would make valuable contributions to the further shaping of patient safety work. This was confirmed during data collection and analysis. The data material drawn from 21 hospital professionals provided a diverse range of information that can be of relevance beyond this specific hospital setting. This enhances the transferability of the results. By eliciting health professionals’ perspectives on how they recognize, respond to, and re-evaluate patients at risk of deterioration with their use of the patient safety package, we believe the results may have transferability beyond the local context where the study was carried out.

We applied a purposive sampling strategy, as proposed by Malterud [39] which contributed with breath in and variety of the data. Our analysis confirmed that the data provided sufficient information power according to the concept of Malterud to bring forth diverse context-rich descriptions to our aim [45]. The study included nurses and physicians from general medical and surgical wards as well as members of the RRT. Their clinical work experiences ranged from sixth months to more than 20 years. The lower end can be a limited time to become completely embedded in practicing patient safety work. However, this implies that the experience of newly employed nurses and physicians was included and compared to more experienced participants**,** representing a breadth in work experience. None of the participants dropped out of the study. Nearly equal numbers of participants from hospital wards and the RRT were included, i.e., 12 and 9, respectively. However, only 4 of the participants were physicians, while 17 were nurses. Thus, the nurses’ perspectives are particularly illuminated in this study. While the physicians’ perspective might not be as prominent as the nurses’ due to a smaller number of participants, the sample accurately represents the distribution of health professions within hospital settings. We obtained a good selection of participants for in-depth interviews with varied experiences, which we consider to be a positive aspect of the study.

## Conclusions

This study highlights the complexity of patient safety work in a hospital setting. The safety package adds structure to the work. In addition, the safety package strengthens and empowers the health professionals in clinical practice and may enable them to recognize patients at risk of deterioration. Furthermore, it provides increased patient safety for the hospital. The study also indicates that focusing on patient safety may contribute to increased collaboration across wards and higher treatment levels, clinical skills, and improved dialogue and judgement within the extended team, around the patient’s bed. Accordingly, the safety package may be embraced to bridge competence gaps that are experienced and are an opportunity to create a learning environment across health professionals which may increase the value of the patient safety work.

### Implications for policy and practice

Norway is among the countries in Europe with the fewest hospital beds per capita and registers a significant percentage of overoccupancy in that respect [9]. Studies show that high occupancy reduces the quality of patient care and increases the incidence of hospital infections and mortality [5, 7]. Therefore, to counteract this development, we suggest that hospital leaders exercise their important role as facilitators and motivators to ensure that the patient safety work is maintained and strengthened in hospitals. Hospital leaders and managers play crucial roles in fostering an empowering environment for their staff within this organization.

Application of the patient safety package can be assessed as the engine of patient safety work at the hospital. This study highlights the importance of a common clinical language, together with the necessary training of the team, to build competence for health professionals to recognize, initiate adequate measures and conduct follow-up response to a deteriorating patient. Variation in patient safety performance might be more related to the complexity of the work at the hospital, and the leaders need to administer and follow up the patient safety package. The importance of facilitating patient safety refresher courses and recertifications for the health professionals should be a priority amongst hospital leaders. It is essential that the professionals maintain and update their level of competence in patient safety work.

## Supplementary Information

Supplementary Material 1.

## Data Availability

Availability of data and materials The data material generated and/or analyzed in this study are not public available from the corresponding authors due to local ownership of data. Aggregated data are available from the corresponding author upon reasonable request.

## Abbreviations

RRSs: Rapid Response System/Systems
RRTs: Rapid Response Team/Teams
METs: Medical Emergency Team/Teams
proACT: Proactive course
NEWS: National Early Warning Score
ABCDEF: Airway, Breathing, Circulation, Disability, Exposure, Further care
ISBAR: Identification (Identify yourself and the patient), Situation (Describe the situation), Background (Provide any relevant background information), Assessment (give your assessment of the situation), Recommendation (What action and further plan or advice do you recommend)
